# Short- and Long-Term Effects of Sodium Phenylbutyrate on White Matter and Sensorimotor and Cognitive Behavior in a Mild Murine Model of Encephalopathy of Prematurity

**DOI:** 10.3390/ijms262412099

**Published:** 2025-12-16

**Authors:** Marie-Anne Le Ray, Cyann Larralde, Lou Legouez, Stéphane Marret, Jean-Baptiste Muller, Bruno J. Gonzalez, Carine Cleren

**Affiliations:** 1 Univ Rouen Normandie, Inserm U1245, F-76000 Rouen, France; marie-anne.le-ray4@univ-rouen.fr (M.-A.L.R.); cyann.larralde@yahoo.com (C.L.); lou.legouez@univ-rouen.fr (L.L.); stephane.marret@chu-rouen.fr (S.M.); jean-baptiste.muller@chu-rouen.fr (J.-B.M.); bruno.gonzalez@univ-rouen.fr (B.J.G.); 2 Univ Rouen Normandie, Inserm U1245 and CHU Rouen, Department of Pédiatrie Néonatale et Réanimation, F-76000 Rouen, France

**Keywords:** perinatal asphyxia, encephalopathy of prematurity, white matter injury, oligodendrocyte, sodium phenylbutyrate, sex, neurobehavior, neonatal care, mice

## Abstract

Perinatal asphyxia (PA) remains a common cause of neonatal death and long-term disability, with an incidence of 20 per 1000 live births. Even mild PA, without significant neurological distress at birth, is linked to neurodevelopmental disorders. Premature babies are at high risk for both PA and long-term neurobehavioral deficits. The use of peripherally inserted central venous catheters in neonatal intensive care units has reduced mortality and morbidity in preterms. Given their prevalent use and associated complications, such as thrombosis, the present study aimed to investigate the effects of hypoxia associated with the ligation of the external jugular vein (JH model) in 5-day-old mice, whose central nervous system development shares similarities with that of human preterms. Diffuse white matter (WM) injury is associated with later neurodisabilities following very premature birth before 32 weeks of gestation. The present study aimed to investigate whether the murine JH model replicates a key phenotype of non-cystic WM injury, namely permanent hypomyelination and sensorimotor deficits. The second aim was to determine whether sodium phenylbutyrate (PBA), which is already prescribed in neonates for another indication, could prevent these disabilities. JH induced lasting dysmyelination in males, not prevented by PBA, contrary to the discrete JH-induced neurobehavioral deficits observed in both sexes in the short and long term.

## 1. Introduction

Encephalopathy of prematurity is characterized by a set of brain lesions associated with interruptions in normal brain maturation in premature infants, later associated with permanent motor, cognitive, and behavioral deficits. The most important lesions are localized in white matter (WM) due to its particular vulnerability between 24 and 32 weeks of gestation [1]. However, due to improved neonatal care in recent decades, there has been a decrease in the proportion of cystic lesions resulting from a non-selective necrotic death in favor of more diffuse ischemic/inflammatory lesions due to the selective death of astrocyte and oligodendrocyte (OL) precursors and a disruption in differentiation processes [2,3]. Globally, this evolution leads to finer deficits but belated neurodevelopmental disorder and medical care, with patients and families suffering from an impacted daily life. This underlines the need to create new animal models, presenting more moderate cerebral lesions associated with long-term sensorimotor, cognitive, and behavioral deficits, such as the model proposed in the present study, the Jugular Hypoxia (JH) model. The JH model was used in 5-day-old (P5) male and female mice. Indeed, at P5, white-matter vulnerability in the rodent would correspond to that observed in preterm human infants [4]. Moreover, it is essential to tend to reproduce the higher vulnerability observed in boys [5].

One aim with the JH model was to reproduce the hypoxic component of cerebral lesions that can be found in extremely premature infants due to the immaturity of their organs, particularly the lungs [6,7]. Indeed, the pulmonary alveoli are not yet fully formed, and surfactant production has not yet stabilized. The resulting hypoxemia can lead to long-term brain damage [8]. Affected children may undergo invasive procedures necessary for neonatal intensive care. Among these procedures, the placement of catheters required for parenteral treatments and/or blood parameter monitoring may lead to complications. Indeed, insertion failures, catheter misplacement, thrombosis, or infections may occur [9]. The risk of thrombosis increases with neonatal hypoxia and low birth weight and is inversely proportional to gestational age. This is because these children have smaller veins, unstable hemodynamics, and a tendency to hypercoagulate [10]. The JH model presented in this study takes that into account. Concretely, it considers three components (prematurity, hypoxia, and neonatal care complications) since it is performed in anesthetized male and female P5 mice undergoing permanent ligation of the left external jugular vein associated with a single episode of hypoxia (45 min, 8% O_2_). The first aim of this study was to evaluate the effects of the JH model on cerebral WM and on mice neurobehavioral abilities, in the short and long term.

Sodium phenylbutyrate (PBA) is a low-molecular-weight fatty acid approved for clinical use as an ammonia scavenger in children and neonates to treat urea cycle disorders [11,12]. PBA can cross the blood–brain barrier and penetrates well into cerebrospinal fluid. It has demonstrated neuroprotective properties in diverse rodent models of perinatal brain lesions. Indeed, PBA inhibits endoplasmic reticulum stress [13] and has anti-apoptosis [14], anti-excitotoxic [15], anti-inflammatory [16], and antioxidant activities [17]. Moreover, recently, PBA was shown to improve MgSO_4_-induced neuroprotection in a neonatal murine model of hypoxia–ischemia which induces severe cerebral lesions [18]. Given these features, it now appears necessary to determine whether PBA may be a good and sufficient therapeutic option for the prevention of encephalopathy of prematurity in a model which tends to mimic current clinical situations. Therefore, the second aim of this study was to evaluate the proper effects of PBA in control mice and its potential neuroprotective effects in the JH model, in the short and long term, while considering sexual differences. For this purpose, sensorimotor abilities were explored in pups (P6–P10), and lesioned cerebral area and OL precursor density were quantified by targeting the corpus callosum and striatum. Indeed, the dorsal striatum, rich in white matter tracts, is involved in sensory and motor functions. It is the input nuclei of basal ganglia receiving incoming information from the cortex, substantia nigra, and thalamus and it is notably capable of action selection, allowing for fine motor control [19]. When adolescence was reached (P30–P45), gross and fine motor abilities were evaluated, as well as cognitive performances such as long-term memory and social behavior, and Myelin Basic Protein (MBP) bundle density was quantified.

## 2. Results

### 2.1. Short-Term Weight Monitoring (Figure S1)

The effects of JH and PBA injection on pups’ weight gain were studied from P5 (before surgery) to P10. At no point did the three-way ANOVA test (Sex; Treatment; Surgery) reveal any differences between sexes. Therefore, sexes were pooled. At no point was there an interaction between the Treatment and Surgery factors (P5: F = 1.584, *p* = 0.234; P6: F = 0.702, *p* = 0.42; P7: F = 0.127, *p* = 0.728; P8: F = 0.813, *p* = 0.387; P9: F = 1.507, *p* = 0.245; P10: F = 1.249, *p* = 0.288).

### 2.2. Short-Term Study of Cerebral White Matter and Tissular Integrity

#### 2.2.1. PDGFRa Immunolabelling (P6)

**Figure 1:**
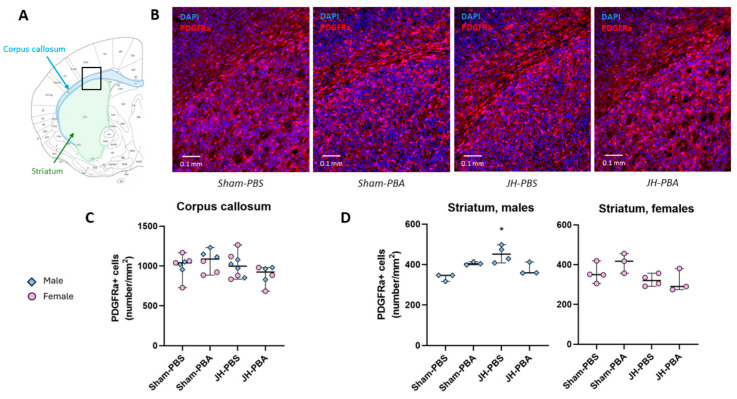
Effects of neonatal JH and/or PBA on PDGFRa cell density in the corpus callosum and striatum at P6. (**A**) Location of the CC and striatum in a P5 mouse brain at 3.27 mm from Bregma [20]. (**B**) Illustrations of PDGFRa immunolabelling in portions of the CC and striatum. (**C**) Quantification of PDGFRa+ cell density in the CC. In the absence of sexual differences, sexes were pooled. (**D**) Quantification of PDGFRa+ cell density in males and females’ striatum. Data are expressed as median ± extreme values. A Kruskal–Wallis test was performed followed by Dunn’s post-test with Bonferroni correction. (* *p* < 0.05 as compared to Sham-PBS male mice). *n* = 6–7 mice/group when sexes were pooled. Statistical details are provided in Table S1.

To assess the short-term impact of JH and PBA injection, alone or in combination, on the OL precursors, OPC, and pre-OL, PDGFRa (Platelet-derived growth factor alpha)-positive cell density was quantified in the ipsilateral hemisphere, 24 h after treatment and surgery (Figure 1). In the CC, a Kruskal–Wallis test did not reveal any difference between sexes. Therefore, data from both sexes were pooled, and no difference between groups was observed (H = 4.96; *p* = 0.18; Figure 1C). In the striatum, a Kruskal–Wallis test revealed a significant difference between groups in males (H = 9.45; *p* = 0.024) and in females (H = 5.41, *p* = 0.015). In males only, the Dunn post hoc test showed that the density of PDGFRa+ cells was significantly greater in JH-PBS pups than in Sham-PBS pups (*p* = 0.015, +34%). Moreover, PBA did not significantly counteract this JH effect (JH-PBA vs. JH-PBS, −17%, *p* = 0.37, Figure 1D). Regarding PBA, it did not show any significant proper effect (Sham-PBA vs. Sham-PBS, *p* = 0.37).

#### 2.2.2. TTC Staining (P10)

**Figure 2:**
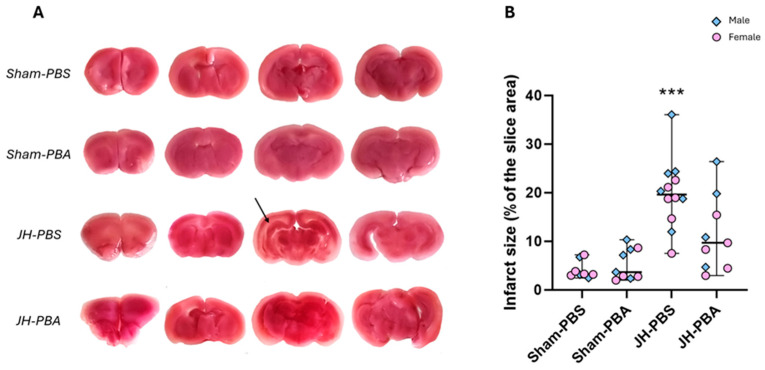
Effects of the neonatal JH and PBA administration on pups’ brain tissular viability at P10. (**A**) Illustration of TTC staining on coronal brain slices from P10 pups. The arrow points to a diffuse WM lesion. (**B**) Quantification of the lesioned area. The sexes are pooled. Data are expressed as median ± extreme values. A Kruskal–Wallis test was performed followed by Dunn’s post-test with Bonferroni correction. (*** *p* < 0.001 as compared to Sham-PBS mice). *n* = 8–12 mice/group when sexes are pooled. Statistical details are provided in Table S1.

**Figure 3:**
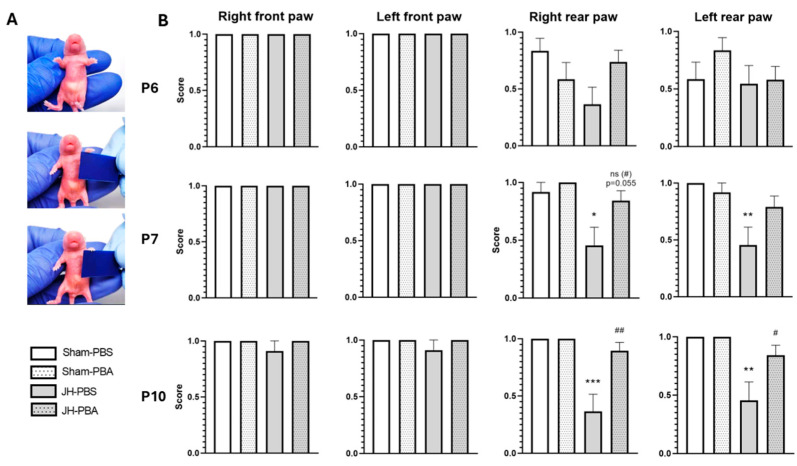
Effects of neonatal JH and PBA administration on pups’ performance in the grasping reflex at P6, P7, and P10. (**A**) Illustrations of the grasping reflex test; (**B**) quantification of the grasping ability (score 1 = success, score 0 = failure). For visibility purposes, results are presented through histograms. The sexes are pooled. Data are expressed as median ± extreme values. A Kruskal–Wallis test was performed followed by Dunn’s post-test with Bonferroni correction. (* *p* < 0.05; ** *p* < 0.01; *** *p* < 0.001 as compared to Sham-PBS; # *p* < 0.05; ## *p* < 0.01 as compared to JH-PBS; ns (#): not significant). *n* = 11–19 mice/group when sexes are pooled. Statistical details are provided in Table S1.

The TTC metabolic test was performed on fresh brain slices to assess the effects of neonatal JH and PBA on tissue viability (Figure 2). There was no statistical difference between sexes (H = 1.89; *p* = 0.169), so sexes were pooled. A statistically significant difference between groups was assessed by a Kruskal–Wallis test (H = 22.1; *p* = 0.00006). The Dunn post hoc test showed that neonatal JH induced tissular loss (JH-PBS vs. Sham-PBS; H = −3.98; *p* = 0.0004; +385%) which was not significantly counteracted by PBA (JH-PBA vs. JH-PBS; H = 1.81; *p* = 0.42), even if PBA reduced the JH-induced lesion area by 43% (Figure 3B). Regarding PBA, it did not show any significant proper effect on the infarct size (Sham-PBA vs. Sham-PBS; H = −0.244; *p* = 1).

### 2.3. Short-Term Study of Behavior

As neurodevelopmental sensorimotor disorders are a major aspect of encephalopathy of prematurity, the aim of these behavioral tests was to assess the short-term impact of JH and PBA administration on sensorimotor abilities.

#### 2.3.1. Grasping Reflex Test (P6, P7, and P10)

This test was carried out to assess JH and PBA effects on spinal reflexes and, therefore, the progressive maturation of pyramidal tracts (Figure 3). At the three times studied, the Kruskal–Wallis test did not reveal any sex effects. Therefore, sexes were pooled. The grasping reflex was fully efficient from P6 to P10 in the four groups in the right front paw P6: *p* = NA; P7: *p* = NA; P10: *p* = 0.27) and in the left front paw (P6: *p* = NA; P7: *p* = NA; P10 = 0.27; Figure 3B). At P6, statistical tendencies underline the fact that the reflex was not yet fully efficient, and there was no difference between the groups in both rear paws (P6 right rear: H = 6.46, *p* = 0.091 P6 left rear (H = 2.74, *p* = 0.434)). At P7 and P10, Kruskal–Wallis tests revealed that there were differences between groups in rear paws (P7 right rear paw: H = 12.9, *p* = 0.0049; P7 left rear paw: H = 11.8, *p* = 0.008; P10 right rear paw: H = 22.4, *p* < 0.001; P10 left rear paw, P10: H = 15.9, *p* = 0.0012). Dunn’s post hoc tests (JH-PBS vs. Sham-PBS) showed that neonatal JH altered the set-up of the grasping reflex in these rear paws at P7 and P10. JH pups were less efficient than Sham pups in grasping (P7 right rear: *p* = 0.029, −50%; P7 left rear: *p* = 0.008, −55%; P10 right rear: *p* = 0.0003, −64%; P10 left rear: *p* = 0.00308, −55%). Moreover, Dunn’s post hoc test revealed that PBA administration significantly prevented or tended to prevent JH-induced alterations of the grasping reflex (JH-PBA vs. JH-PBS). Indeed, JH pups that received PBA were or tended to be more efficient in grasping than JH pups that received PBS (P7 right rear: *p* = 0.055 ns, +87%; P10 right rear: *p* = 0.0012, +146%; P10 left rear: *p* = 0.039, 85%). Finally, Dunn’s post hoc test revealed that PBA did not present significant proper effects in Sham mice at any of the timepoints (Sham-PBA vs. Sham-PBS at P6, P7, P10 in rear paws: *p* = 1).

#### 2.3.2. Cliff Aversion Test (P6, P7 and P10)

**Figure 4:**
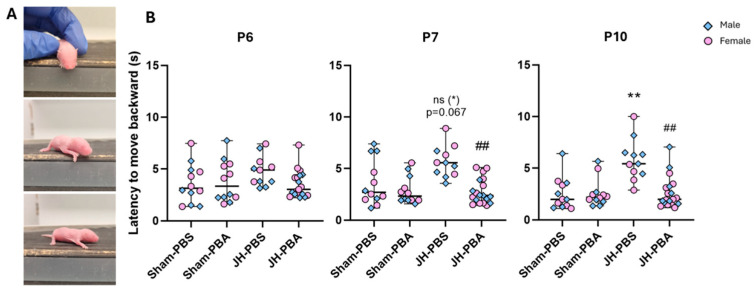
Effects of the neonatal JH and PBA administration on the duration to move backward in the cliff aversion test at P6, P7, and P10. (**A**) Illustrations of the cliff aversion test; (**B**) quantification of the time needed to move backward. Sexes are pooled. Data are expressed as median ± extreme values. The Kruskal–Wallis test was performed followed by Dunn’s post-test with Bonferroni correction. (** *p* < 0.01 as compared with Sham-PBS; ## *p* < 0.01 as compared with JH-PBS; ns (*): not significant). *n* = 11–19 mice/group when sexes are pooled. Statistical details are provided in Table S1.

This test was carried out to assess JH and PBA effects on labyrinth reflexes, coordination, and strength (Figure 4). At P6, a Kruskal–Wallis test showed no differences between groups (P6: H = 5.36, *p* = 0.148), while at P7 and P10, the Kruskal–Wallis tests showed significant differences between groups (P7: H = 16.3; *p* = 0.001; P10: H = 18, *p* = 0.0004; Figure 4B). Dunn’s post hoc tests showed that neonatal JH significantly altered (P10) or tended to alter (P7) pups’ ability to move backward. Indeed, JH pups took more time to move backward than Sham pups (JH-PBS vs. Sham-PBS; P7: *p* = 0.067 ns, +56%; P10: *p* = 0.0015, +108%). PBA administration reduced the duration taken to move backward in JH mice (JH-PBA vs. JH-PBS; P7: *p* = 0.001, −50%; P10: *p* = 0.001, −55%). Moreover, it is noteworthy that JH pups treated with PBA were as efficient as Sham-PBS pups (JH-PBA vs. Sham-PBS; P7: *p* = 1; P10: *p* = 1). Therefore, PBA totally prevented JH-induced deficits at P10. At the three timepoints, no proper effect of PBA was observed. Indeed, Sham pups treated with PBA needed a similar time to move backward than Sham pups (Sham-PBA vs. Sham-PBS; P7: *p* = 1; P10: *p* = 1).

#### 2.3.3. Negative Geotaxis Test (P6 and P7)

**Figure 5:**
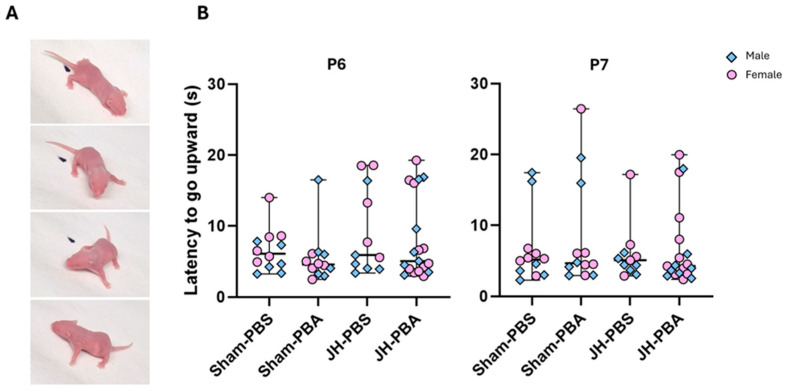
Effects of the neonatal JH and PBA administration on pups’ performance in the negative geotaxis test at P6 and P7. (**A**) Illustrations of the negative geotaxis test; (**B**) quantification of the time needed to move upward. Sexes are pooled. Data are expressed as median ± extreme values. A Kruskal–Wallis test was performed followed by Dunn’s post-test with Bonferroni correction. *n* = 11–19 pups/group when sexes are pooled. Statistical details are provided in Table S1.

This test was carried out to assess JH and PBA effects on pups’ ability to analyze motor and vestibular inputs and on pups’ motor coordination (Figure 5). At P6 and P7, the Kruskal–Wallis tests did not show any significant differences between the sexes, nor in the time needed to go upward between the four groups (P6: H = 2.79, *p* = 0.43; P7: H = 0.585, *p* = 0.9; Figure 5B).

#### 2.3.4. Righting Reflex Test (P6, P7)

**Figure 6:**
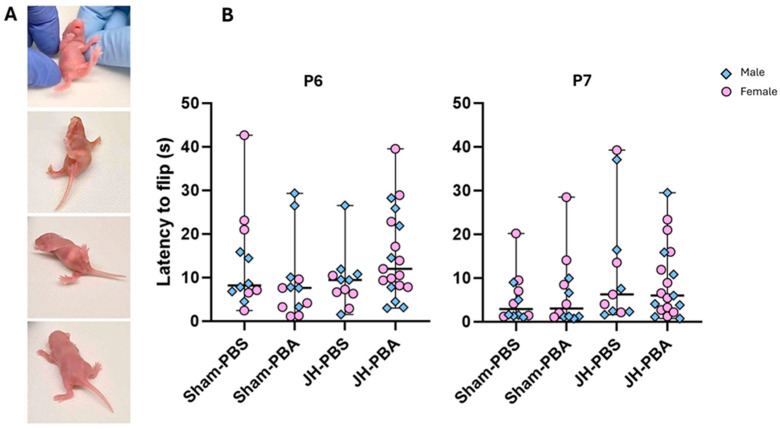
Effects of the neonatal JH and PBA administration on pups’ performance in the righting reflex test at P6 and P7. (**A**) Illustrations of the righting reflex test; (**B**) quantification of the time needed to flip. Sexes are pooled. Data are expressed as median ± extreme values. A Kruskal–Wallis test was performed followed by Dunn’s post-test with Bonferroni correction. *n* = 11–19 pups/group when sexes are pooled. Statistical details are provided in Table S1.

This test was carried out to assess JH and PBA effects on sensorimotor abilities and motor coordination, strength, and vestibular balance, as well as joint and muscle receptor functioning (Figure 6). At P6 and P7, the Kruskal–Wallis tests did not reveal any significant differences between sexes, nor in the time needed to flip between the four groups (P6: H = 5.15, *p* = 0.161; P7: H = 3.96, *p* = 0.266; Figure 6B).

### 2.4. Long-Term Study of White Matter (P45)

**Figure 7:**
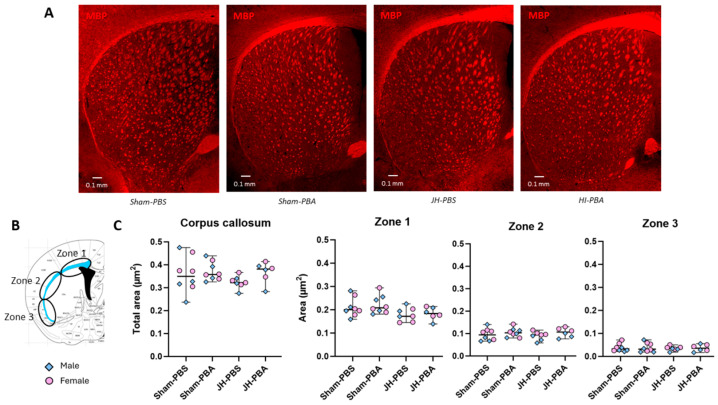
Effects of JH and PBA administration on MBP immunolabelling in the ipsilateral corpus callosum at P45. (**A**) Illustration of MBP immunolabelling in the CC and striatum; (**B**) locations of the CC divided into three zones; and (**C**) quantification of the areas of the CC and its three zones. Data are expressed as median ± extreme values. The Kruskal–Wallis test was performed followed by Dunn’s post-test with Bonferroni correction. *n* = 6–8 mice/group when sexes are pooled. Statistical details are provided in Table S1.

**Figure 8:**
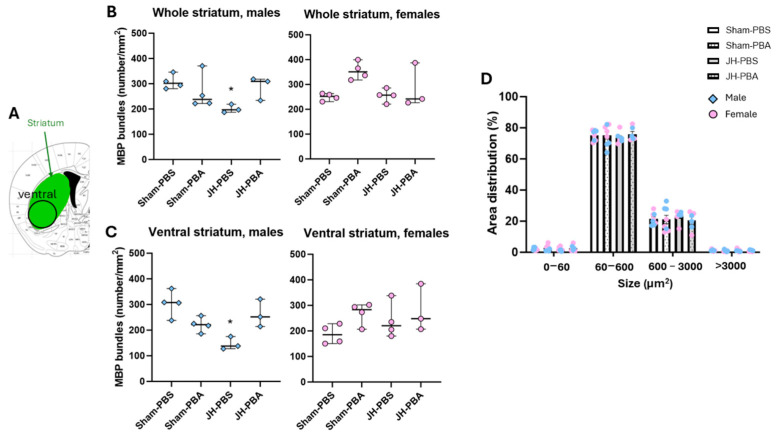
Effects of JH and PBA administration on MBP bundle density in the ipsilateral whole striatum and ventral striatum at P45. (**A**) Location of the whole striatum and the ventral part studied; (**B**) quantification of MBP bundle density in the whole striatum in males and females; (**C**) quantification of MBP bundle density in the ventral striatum in males and females; and (**D**) distribution of MBP bundle area. Bundles are distributed in four classes (µm^2^); Data are expressed as median ± extreme values. For (**B**,**C**), a Kruskal–Wallis test with Bonferroni correction was performed followed by Dunn’s post-test. For (**D**), a Chi-square test was performed. (* *p* < 0.05 as compared to Sham-PBS). *n* = 6–8 mice/group when sexes are pooled. Statistical details are provided in Table S1.

To assess the long-term impact of JH and PBA on mature myelinating OL, MBP labeling was performed forty days after JH (Figure 7 and Figure 8).

#### 2.4.1. Study of White Matter in the Corpus Callosum (Figure 7)

The Kruskal–Wallis test did not show any significant differences between sexes, nor between groups, whether the area of the CC was analyzed as a whole or as three independent zones (total area: H = 6.86, *p* = 0.077; zone 1 area: H = 4.85, *p* = 0.183; Zone 2 area: H = 2.68, *p* = 0.444; zone 3 area: H = 0.514, *p* = 0.916; Figure 7C).

#### 2.4.2. Study of White Matter in the (Ventral) Striatum (Figure 8)

A Kruskal–Wallis test revealed significant differences in males regarding the density of MBP bundles in the whole striatum (Figure 8B), as well as in the ventral striatum (bundle density in the whole striatum: H = 9.03, *p* = 0.0289; bundle density in the ventral striatum: H = 8.91, *p* = 0.031; Figure 8C). Dunn’s post hoc tests revealed that, in males, neonatal JH significantly reduced the density of MBP bundles in the whole striatum, as well as in the ventral striatum (JH-PBS vs. Sham PBS; bundle density in the whole striatum: *p* = 0.034, −35%; bundle density in the ventral striatum: *p* = 0.023; −52%). Moreover, Dunn’s post hoc test revealed that PBA did not significantly counteract JH-induced reductions in bundle density in males. Indeed, male JH pups treated with PBA showed a non-significant increase in bundle density (JH-PBA vs. JH-PBS; bundle density in the whole striatum: *p* = 0.13 ns, +43%; bundle density in ventral striatum: *p* = 0.2 ns, +79%).

To end, there was no proper effect of PBA on MBP bundle density (Sham-PBA vs. Sham-PBS; bundle density in the whole striatum: *p* = 0.39; Figure 8B; bundle density in the ventral striatum: *p* = 0.51; Figure 8C). Moreover, there was no effect of neonatal JH or PBA administration on the distribution of MBP bundle area in the whole striatum (Chi square = 8.653; *p* = 0.470; Figure 8D).

### 2.5. Long-Term Study of Behavior

#### 2.5.1. Social Approach and Memory Test (P30 and P31)

**Figure 9:**
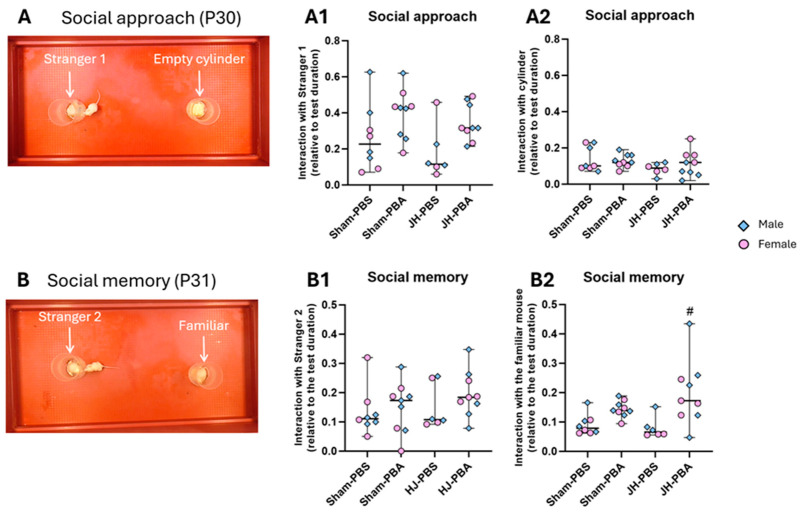
Effects of neonatal JH and PBA administration on social behavior in adolescent mice. (**A**) Illustration of the social approach phase of the test (P30) and quantification of interactions duration with Stranger 1 and with the empty cylinder (relative to the test duration); (**B**) illustration of the social memory phase of the test (P31) and quantification of interactions duration with Stranger 2 and with the familiar mouse (relative to the test duration). Sexes are pooled. Data are expressed as median ± extreme values. A Kruskal–Wallis test was performed followed by Dunn’s post-test with Bonferroni correction. (# *p* < 0.05 as compared with JH-PBS). *n* = 6–9 mice/group when sexes are pooled. Statistical details are provided in Table S1.

This test evaluates the long-term impact of JH and PBA on social abilities (Figure 9). The tested mouse was placed in the following two different situations: first at P30 with a stranger mouse, then at P31 with two mice, one familiar and one unknown. For the social approach, the Kruskal–Wallis test revealed that there was a significant difference between groups with regard to the duration of the interaction of the tested mice with Stranger 1 (H = 8.26, *p* = 0.041; Figure 9(A1)), while there was no difference between groups with regard to the duration of the interactions with the empty cylinder (H = 3.68, *p* = 0.298; Figure 9(A2)). For social memory, the Kruskal–Wallis test did not reveal any differences between groups in terms of the duration of interactions with Stranger 2, which was the new mouse (H = 2.992, *p* = 0.393; Figure 9(B1)). However, in terms of the duration of interaction with the familiar mouse (ex-Stranger1), groups differed (H = 12.81, *p* = 0.0051; Figure 9(B2)). Dunn’s post hoc test revealed that JH-PBA mice interacted more with Stranger 2 than JH-PBS mice (*p* = 0.02).

#### 2.5.2. Balance Beam Test (P32)

**Figure 10:**
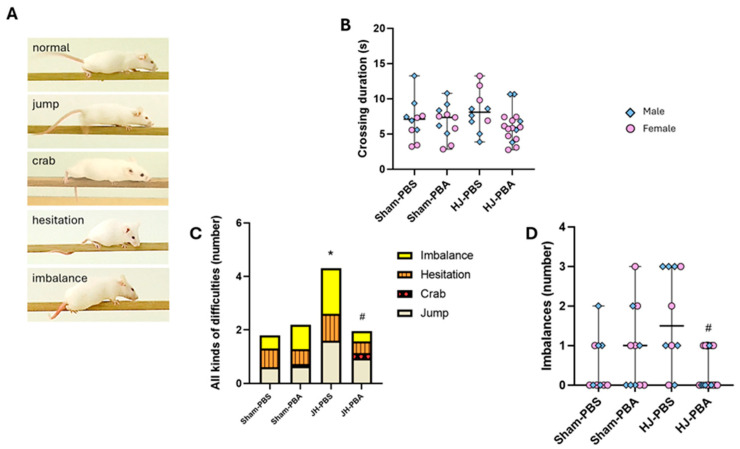
Effects of neonatal JH and PBA administration in the balance beam test in adolescent (P32) mice. (**A**) Illustration of normal locomotion and different difficulties encountered by mice while crossing the beam; (**B**) quantification of the time required to cross the beam; (**C**) quantification of the total number of all different kinds of difficulties (the proportion of each difficulty is illustrated within the bars of the graph); and (**D**) quantification of the number of imbalances. Sexes are pooled. Data are expressed as median ± extreme values. A Kruskal–Wallis test was performed followed by Dunn’s post-test with Bonferroni correction. (* *p* < 0.05 as compared with Sham-PBS; # *p* < 0.05 as compared with JH-PBS. *n* = 10–16 mice/group when sexes are pooled. Statistical details are provided in Table S1.

This test assesses the long-term impacts of JH and PBA on fine motor coordination and balance (Figure 10). Concerning the time taken by mice to cross the beam, a Kruskal–Wallis test did not reveal any differences between groups (H = 4.162, *p* = 0.244; Figure 10B). Concerning the total number of different difficulties encountered by mice while crossing the beam, the Kruskal–Wallis test revealed that groups differed (H = 11.29, *p* = 0.0103; Figure 10C). The Dunn’s post hoc test revealed that JH-PBS mice presented more difficulties than Sham PBS mice (*p* = 0.038) and that PBA significantly prevented the JH-induced increase in the number of difficulties (JH-PBA vs. JH-PBS, *p* = 0.0102). Since JH-PBA mice did not differ from Sham-PBA mice (*p* = 1), this prevention was complete. PBA did not show any proper effects (Sham-PBA vs. Sham-PBS, *p* = 1). The Kruskal–Wallis test also revealed that the number of imbalances differed between groups (H = 9.21, *p* = 0.0266; Figure 10D). Dunn’s post hoc test revealed that JH did not significantly increase the number of imbalances (JH-PBS vs. Sham-PBS, + 240%) but that JH-PBA mice presented fewer imbalances than JH-PBS (*p* = 0.0245). Moreover, PBA did not present significant proper effects (Sham-PBA vs. Sham-PBS, *p* = 1).

#### 2.5.3. Foot-Fault Test (P33)

**Figure 11:**
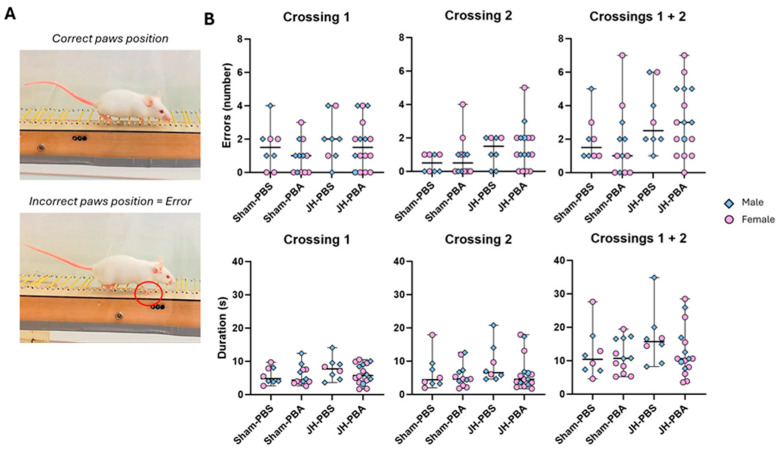
Effects of neonatal JH and PBA administration in the Foot-Fault Test at P33. (**A**) Illustration of correct and incorrect paw positions; (**B**) effect of neonatal JH and PBA on gait in adolescent male and female mice for the first (crossing 1), the second (crossing 2), and both crossings (crossing 1 + 2). The sexes are pooled together. Data are expressed as median ± extreme values. The Kruskal–Wallis test was performed followed by Dunn’s post-test with Bonferroni correction. *n* = 10–16 mice/group when sexes are pooled. Statistical details are provided in Table S1.

This test was used to study the long-term impacts of JH and PBA on the mouse’s ability to adapt its gait according to the environmental context, knowing that gait is influenced by the temporal and spatial integration of the cognitive and neuromusculoskeletal neural systems (Figure 11). For the first (Crossing 1), the second (Crossing 2), and both crossings (Crossings 1 + 2), the Kruskal–Wallis test did not show differences between the groups concerning the number of errors (first: *p* = 0.3; second: *p* = 0.13; both *p* = 0.1; Figure 11B) and the duration to cross (first: *p* = 0.5; second: *p* = 0.26; both: *p* = 0.49). It is noteworthy that some mice seemed to improve their performance (number of errors) during the second crossing, while others seemed to perform worse.

#### 2.5.4. Novel Object Recognition Test (P34)

**Figure 12:**
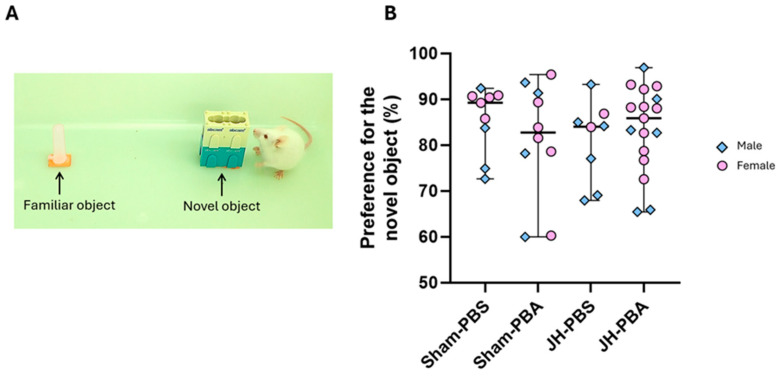
Effects of neonatal JH and PBA administration in the Novel Object Recognition Test at P34. (**A**) Illustration of the second phase of the NOR test; (**B**) quantification of the preference for the new object as opposed to the familiar one. The sexes are pooled. Data are expressed as median ± extreme values. A Kruskal–Wallis test was performed followed by Dunn’s post-test with Bonferroni correction. *n* = 8–17 mice/group when sexes are pooled. Statistical details are provided in Table S1.

This test was used to assess long-term impact of JH and PBA on learning and memory (Figure 12). The Kruskal–Wallis test did not reveal any differences between the four groups concerning preference for the novel object as compared to the familiar one (H = 1.06, *p* = 0.786).

## 3. Discussion

Premature babies are at high risk for perinatal hypoxia. Neonatal adaptation is often complicated by the necessity for resuscitation at birth. Indeed, more than 50% of premature infants suffer from episodic hypoxemic events, with or without associated bradycardia, including when they are on non-invasive ventilation [21]. Among survivors, approximately 25% experience permanent neurologic deficits and 15–30% develop cognitive impairments, speech difficulties, and behavioral disorders, even in the absence of severe neurological disability. Indeed, even mild to moderate perinatal hypoxia, without significant neurological distress at birth, is linked to neurobehavioral developmental disorders, i.e., motor, cognitive, and communication deficits [22].

Hypoxia caused by underdeveloped neural vasculature and inefficient oxygenation from immature lungs is thought to play a significant role in cerebral WM injury, because susceptibility to diffuse WM injury occurs before the onset of myelination, when the most prevalent members of the OL lineage are OL precursors [23]. The recent evolution of WM injury pathology from cystic PVL toward diffuse hypomyelinating disease suggests a mechanistic shift toward dysfunction of myelinating cells. This emerging pattern of non-cystic WM injury requires animal models in which signs of cellular demise and degeneration are minimal, but a failure of postnatal brain myelination is prominent, associated with long-term neurobehavioral deficits [24].

The first aim of this study was to obtain a preterm model that leads to long-term diffuse WM injury associated with motor and cognitive deficits, which could be transposed to clinical practice. For this purpose, an acute and relatively short hypoxia was applied to P5 mice, when OL precursor differentiation was ongoing and OL myelination had barely started in the murine brain [25]. Moreover, the external jugular vein was ligated in order to take into account the fact that preterm neonates often undergo invasive procedures such as the placement of catheters for parenteral treatments and/or blood parameter monitoring, which can lead to complications such as thrombosis or infections [9]. Indeed, the risk of thrombosis increases with neonatal hypoxia and is inversely proportional to gestational age. Altogether, this procedure led to the Jugular Hypoxia (JH) model. The second aim of this study was to investigate whether sodium phenylbutyrate alone could prevent the potential deficits induced by the JH model, due to its reported neuroprotective properties [14,15,26].

Short-term exposure to low oxygen levels is known to induce mild to moderate cell damage in the neonatal rodent brain [27]. The present study reports that JH altered cerebral tissue viability in both male and female pups, as observed five days after injury. TTC-stained slices showed that different regions of the brain had variable sensitivity to hypoxia. This could be linked to the different levels of oxygen delivery required [21]. In response to acute hypoxia, selectively vulnerable regions in the developing mouse brain, mainly the striatum, can undergo continued apoptosis for a prolonged period up to 6–7 days post insult [28].

When focusing on WM, the JH model led to short- and long-term alterations in the striatum, but only in males. Indeed, as soon as twenty-four hours after injury, the density of OL precursors increased in the male striatum, as reported by the Cellpose trained model (which reduces counting bias). In the same vein, forty days after injury, male JH mice showed a lower density of myelinated fibers in their striatum. This phenomenon was amplified in the ventral part of the striatum. The striatum is notably involved in sensory and motor functions. It is capable of action selection, allowing fine motor control [19]. More OL precursors but fewer MBP-expressing cells (mature myelinating OL) could reflect the presence of OL precursors trapped in the cell cycle and/or cell death occurring in mature OL [21]. Disturbed myelination (hypomyelination), resulting from arrested OL precursor differentiation, is one of the established pathological hallmarks in the brain of preterm neonates affected by diffuse WM injury [25]. Regarding sexual differential vulnerability, it may result from the earliest sexual brain differentiation in male pups. In males, a peak of testicular testosterone production occurs in the last few days of gestation and again approximately 2 h after birth, while circulating testosterone remains low in females during the perinatal period [5,29].

When focusing on WM within the CC, the density of OL precursors and mature OL was not impacted in either males or females. A study reported hypomyelination in CC in mice after chronic intermittent (11% O_2_, 4 min cycles, 4 weeks) or continuous (11% O_2_, 2 weeks) hypoxia starting at P2 [30]. In three intermittent hypoxia models mimicking hypoxia/reoxygenation events occurring in infant apnea, Darnall et al. (P2–P16) in rats and Juliano et al. (P1–P15) and Cai et al. (P2–P16) in mice found biochemical and electron microscopy evidence of impaired axonal myelination in the CC [21,24,31]. The absence of a JH-induced deleterious effect in the CC in the present study might be due to a too-short episode of hypoxia (45 min, 8% O_2_), an insufficiently resolutive method to observe it, the delayed myelination of the CC compared to that of the striatum during mouse development [32], or to the absence of repetitive phases of reoxygenation. An extensive review of the literature conducted for the present study reports that no study has investigated acute and short hypoxia effects in P5 mice or in P3 rats (equivalent to P5 mice [33]). Therefore, the JH model could help refine commonly used methods when targeting discrete deficits in accordance with the 3Rs rule.

In human neonates, symptoms associated with perinatal asphyxia (e.g., muscular hypotonia and cardiorespiratory failure) may occur within the first hours post injury [27]. In the present study, pups underwent complementary sensorimotor tests starting 24 h post-injury. JH altered pups’ performances in two out of four tests in both sexes, namely the cliff aversion test and the grasping reflex test. Regarding the latter, in both sexes, the deficits induced by JH concerned the rear paws. This could be explained by the timing of myelination progression within the body, with the nerves of the front paws being myelinated earlier than those of the rear paws. Consequently, JH seems to impair or at least delay the maturation of corticospinal tracts. Regarding JH-induced deficits in the cliff aversion test, they could result from altered labyrinthine reflexes, since motor coordination and strength seemed unaltered in the two other tests. When analyzed in the long term through the balance beam test, it appeared that this deficit persisted. Indeed, JH mice showed more difficulties and tended to suffer from more imbalances when crossing. It is noteworthy that mice succeeded in crossing the beam without falling and without spending more time than controls. This implies that they deployed strategies to efficiently compensate for their deficits, such as rolling the tail around the beam or walking like a crab. Moreover, other behavioral tests could reveal sex-dependent deficits.

Regarding cognition abilities, JH did not seem to alter social behavior nor learning and memory in the long term. However, Kelemen et al. (2025) [22] reported that short and acute hypoxia in P7 rats slightly decreased social sniffing during the social interaction test. It would be interesting to have JH mice undergo the direct social interaction test while recording their vocalizations. Furthermore, JH mice exhibited seemingly normal growth. This is not surprising, since Cai et al. (2012) [21] reported that there was no difference in body weight in an intermittent hypoxia model lasting from P2 to P10 (8.0% O_2_, 120 s).

Regarding PBA efficiency in JH-induced deficits, it failed to significantly reduce mild to moderate alterations of cerebral tissue and striatal WM, probably due to high interindividual variability. However, it prevented short- and long-term JH-induced behavioral deficits. Altogether, it seems that PBA did not prevent OL precursor accumulation, but could compensate for this by preventing JH-induced delays in myelination, possibly by promoting gene expression through its HDAC inhibitory properties [18,26]. Indeed, DNA methylation, histone modifications, and microRNAs are major contributors to OL precursor differentiation [34]. A regional multi-omics study of PBA effects in JH mice through high-plex spatial protein profiling on brain sections at P6 would allow for identifying the mechanism underlying PBA neuroprotective effects. Besides these effects, it is noteworthy that PBA did not show any proper deleterious effect at the histological level or in the different behavioral tests performed. This study will be part of a whole study of JH molecular mechanisms that will simultaneously test the effects of PBA alone or associated with a second molecule. This will prevent the use of too many mice.

We mentioned in a review the reasons why considering sex effects is necessary [5]. The present study attests to this. The intersexual variability could be attributed, in part, to sexual dimorphism in inflammatory responses and microglial function during the perinatal period [22]. The male CC contains more OL precursors at P5 than the female CC [35]. Moreover, learning and recognition memory have been reported to be compromised following lesions to the CC [36]. JH did not show alterations in either recognition memory performance (NOR test) or CC WM bundle density. Before concluding, it will be necessary to analyze the ultrastructure of WM on sagittal slices of the CC through electronic microscopy. Simultaneously, the effects of PBA alone or associated with a second molecule will be studied. Indeed, a high percentage of unmyelinated axons and axons with thinner myelin sheaths have been reported in neonatal brains after short-term intermittent hypoxia [21], but the density of OL is greater and myelin sheaths are thicker in the male mouse brain [35]. Studying other brain structures and other cell types will be indispensable, since astrocytes, microglial, and endothelial cells are present in the developing WM [37]. Moreover, having mice perform behavioral tests targeting other early and/or discrete alterations, such as pups’ vocalizations or in adult mice, the seed-grasping test would be useful, as it could help identify deficits in females.

### 3.1. Limitations of the Study

The JH model presented in this study induced modest effect sizes, with high interindividual variability. Corpus callosum alterations and cognitive deficits were not reported through the investigated parameters. Molecular studies will have to be performed and PBA effects will have to be improved.

### 3.2. Conclusions

To conclude, the Jugular Hypoxia model could be a valuable model for non-cystic WM injury, at least in males, in which signs of cellular demise and degeneration are mild but the failure of postnatal brain myelination is prominent, associated with long-term behavioral deficits. The JH model presents interindividual and intersexual variabilities, which are in accordance with the clinical situation. This heterogeneity provides a valuable opportunity to investigate individual variability in vulnerability. This variation closely mirrors the clinical diversity seen in human survivors of mild to moderate perinatal asphyxia, many of whom do not show overt structural damage but experience long-term behavioral dysfunction. Regarding PBA, it was able to prevent short- and long-term JH-induced sensorimotor deficits in both sexes, but it did not prevent short- and long-term histological alterations observed in males. This highlights the interest of this molecule in neuroprotection and warrants further investigation of its mechanism of action in mild murine models of encephalopathy of prematurity.

## 4. Materials and Methods

### 4.1. Animals and Housing

The French Ministry of National Education and Research granted authorization for the handling of animals in accordance with the directives of the Council of the European Communities (2010/63/EU) and national laws (APAFIS #38027-2021121714411664 v8). Mice from the National Marine Research Institute (NMRI, Bergen, Norway) were acquired from Janvier laboratory (France). Mice were housed at 22 °C with a 12 h light/dark cycle (lights on from 7 am to 7 pm) and food and water ad libitum. From P5 to P45, they were handled daily. At P21, males and females were weaned and placed in separate cages. At P6, P10, and P45, the mice were euthanized using ethical methods The number of animals was as reduced as possible (Table S1). Animal welfare was ensured throughout the study.

### 4.2. The Jugular Hypoxia Model

**Figure 13:**
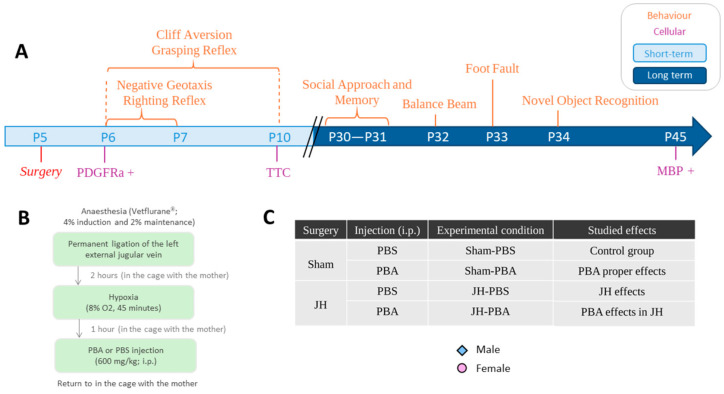
(**A**) Experimental design of the study; (**B**) details of the “Jugular Hypoxia” and treatment procedures; and (**C**) experimental conditions; number of mice/group is detailed in Table S1. Pups from different litters were randomly assigned to different groups, ensuring gender balance. The mice from the different groups underwent the behavioral tests sequentially or randomly. HI: Hypoxia–Ischemia; P5: Postnatal day 5; i.p.: intraperitoneally; PBS: Phosphate-buffered saline; PBA: Sodium Phenylbutyrate; JH: Jugular Hypoxia, O_2_: Oxygen; PDGFRa: Platelet-Derived Growth Factor Receptor Alpha; TTC: Trimethyl Tetradecylammonium Chloride; MBP: Myelin Basic Protein.

To target WM, the procedure was performed in P5 mice (Figure 13). Indeed, at P5, white-matter vulnerability in rodents would correspond to that in humans born between 24 and 32 gestational weeks (GW) [4]. In anesthetized P5 pups (isoflurane 4% for the induction and 2% for the maintenance, VETFLURANE^®^), the left jugular vein was permanently ligated. Two hours after ligation, pups were placed in a hypoxic chamber with 8% oxygen for 45 min at 36 °C. One hour after the end of hypoxia, an intraperitoneal injection (i.p.) of PBA (600 mg/kg) or phosphate-buffered saline (PBS) was performed. The choice of these parameters (duration and O_2_ percentage) originates from a previous study from the laboratory where hypoxia was coupled with the ligature of the carotid [18]. The choice of this PBA dose originates from a previous study from the laboratory [18]. The reasons for choosing the same parameters as previous studies for hypoxia and PBA were (1) to be able to compare these two models in a future study and (2) to test complementary studies on PBA effects at this dose. Sham animals underwent anesthesia and the surgical procedure, but without vein ligation. They were also isolated from their mother and kept at 36 °C in a normoxic environment. The behavior of mothers towards pups was observed before, during, and after surgery to ensure the lowest possible level of stress. Potential repetitive actions such as compulsive cage cleaning, freezing, and disinterest in pups were checked. Pup feeding was also checked, particularly after the ligation, hypoxia, and PBA injection procedures.

### 4.3. Short-Term Weight Monitoring (P5–P10, Figure S1)

The impact of neonatal JH and PBA injection on pups’ weight gain was monitored daily from P5 to P10.

### 4.4. Short-Term Study of White Matter and Cerebral Tissue

#### 4.4.1. PDGFRa Immunolabeling (P6; Figure 2)

To assess the short-term impacts of JH and PBA on the oligodendrocyte (OL) precursors, OPC, and pre-OL, PDGFRa immunolabeling was performed on brain slices. Cell density was evaluated in the corpus callosum (CC) and the striatum, both cerebral structures rich in WM. The striatum is a complex brain area involving sensory and motor functions. It is the input nuclei of basal ganglia receiving incoming information from different cerebral areas and it is notably capable of action selection, allowing for fine motor control. The striatum also deals with emotions and motivational state in mammals [19]. The CC enables the communication between the two cerebral hemispheres and it is involved in learning and recognition memory. For this purpose, 24 h after JH brains were extracted and immersed in 4% paraformaldehyde (PFA) for 1 h, they were then transferred in a cryoprotective solution (sucrose/PBS 30%). They were frozen in isopentane at −30 °C and later stored at −80 °C until 20 µm sections were cut. Sections were incubated for 45 min in a permeabilizing buffer (PBS, BSA 1%, triton X-100 0.3%) at room temperature before being incubated overnight at 4 °C with the primary antibody (1/200 in permeabilizing buffer) directed against PDGFRa (R&D system AF1062, Goat polyclonal, Minneapolis, MN, USA). The next day, after 3 rinses of 10 min each in permeabilizing buffer, these sections were incubated for 1.5 h in the dark with slow shaking, with the secondary antibody (1/400 in permeabilizing buffer) Alexa Fluor 594 Donkey anti-goat (Life technology A11058, Waltham, MA, USA). After 3 rinses of 10 min each in PBS, the slices were incubated with a fluorescent DNA intercalating agent to label nuclei (Hoechst, 1/5000 in PBS, Sigma Aldrich, Saint Louis, MO, USA). Slices were observed by using the Leica DMI 6000B microscope and the images were obtained using the Metamorph^®^ Leica AF software (7.8; Molecular Devices, San Jose, CA, USA). For a quantitative evaluation of PDGFR immunofluorescence, the density (number/mm^2^) of PDGFRa-positive (PDGFRa+) cells in the corpus callosum (Bregma: 3.27 mm) and in the striatum were measured using a trained Cellpose model and ImageJ Fiji software (1.54p, 64 bit; National Institute of Health, Bethesda, Rockville, MD, USA). The Cellpose model was trained on 4410 ROIs and was evaluated at 96% accuracy. For each animal, the density of PDGFRa+ cells was assessed by averaging two successive brain sections.

#### 4.4.2. TTC Staining (P10; Figure 3)

The TTC metabolic test, allowing us to assess tissue viability, was performed on brain slices from P10 mice. On ice, fresh brains were sectioned into 1 mm thick slices in a coronal plane using a brain slicer (Zivic Labs). Slices were immediately immersed in TTC 2% (Sigma, 93140-50G) in the dark at room temperature for 30 min, followed by paraformaldehyde (PFA) 4% at 4 °C for at least 2 h. Lesioned areas were quantified on acquired images using the ImageJ Fiji software.

### 4.5. Short-Term Study of Sensorimotricity

#### 4.5.1. Grasping Reflex Test (P6, P7, and P10; Figure 4)

In pups, this reflex promotes maternal cares, allowing the pup to grasp its mother’s fur. This test was carried out at P6, P7, and P10 to assess forepaw or hindpaw issues. This test reveals spinal reflexes and, therefore, the progressive maturation of pyramidal tracts [38]. Each paw was tested by touching it with a non-sharp but thin object. The presence or the absence of grasping was recorded with a score of 1 when the pup presented the grasping reflex and a score of 0 when it did not [39].

#### 4.5.2. Cliff Aversion Test (P6, P7, and P10; Figure 5)

In pups, this reflex promotes survival, preventing them from falling. This test was carried out at P6, P7, and P10 to assess labyrinth reflexes via vestibular imbalances, coordination, and strength. Since at this age, pups’ eyes are still closed, it is not sight but the sensation of emptiness that motivates them to move away from the void [21]. Pups were placed on a 17 cm high box with their muzzle and front paws overhanging the void. To turn their muzzle away from the void, they had to use their front and hind legs. The time taken to turn around was assessed.

#### 4.5.3. Negative Geotaxis Test (P6 and P7; Figure 6)

Motor and vestibular input are required for the mouse to recognize its orientation on a slope and turn around. This test was carried out at P6 and P7 to assess vestibular and motor coordination, especially of the forepaws [39]. For this purpose, pups were placed facing down on the support, which was tilted at 30°, and had to turn around to position their heads upwards. The time needed for a pup facing downhill on a sloped surface to turn and face up the slope was recorded. Each value corresponds to the mean of 2 trials, separated by a 10 s delay. The cut-off time per trial was set at 60 s.

#### 4.5.4. Righting Reflex Test (P6, P7; Figure 7)

The righting reflex is the motor ability of a mouse pup to be able to flip onto its four feet from a supine position. This test was carried out at P6 and P7 to assess sensorimotor abilities, as well as motor coordination, strength, vestibular balance, and joint and muscle receptor function [39]. The time needed for a pup to turn over was recorded. Each value is the mean of 3 successive trials separated by a 10 s delay. The cut-off time per trial was set at 60 s.

### 4.6. Long-Term Study of White Matter

#### MBP Immunolabeling (P45; Figure 8 and Figure 9)

To assess the long-term impacts of JH and PBA on WM, MBP immunolabeling was performed 40 days post JH (P45). Brains were prepared using a protocol similar to that presented for the PDGFRa-labeled short-term immunofluorescence study. The brains were cut into 40 µm sections and incubated first with the primary antibody MBP (Abcam Ab7349, Rat monoclonal, Cambridge, UK) and then with Alexa Fluor 594 Donkey anti-Rat (Invitrogen A-21209, Waltham, MA, USA). Slices (Bregma: 0.02 mm) were observed using the Leica DMI 6000B microscope and the images were obtained using the Metamorph^®^ Leica AF software (7.8; Molecular Devices, San Jose, CA, USA). Using a Cellpose trained model and ImageJ software (1.54p, 64 bit; National Institut of Health, Bethesda, Rockville, MD, USA), the corpus callosum (CC) area, total number, density (number/mm^2^), and the size of striatum fiber bundles were measured in the ipsilateral hemisphere. The CC was divided into three zones, where the first zone (zone 1) corresponds to axons mainly projecting to the motor cortex, the second zone (zone 2) to those mainly projecting to the somatosensory cortex, and the third zone (zone 3) to those projecting mainly to the primary and associative auditory cortex [40]. The Cellpose model was trained on 5497 ROIs and was evaluated at 99% accuracy.

### 4.7. Long-Term Study of Sensorimotor and Cognitive Abilities

#### 4.7.1. Social Approach and Memory Test (P30 and P31; Figure 10)

This two-phase test mobilizes the natural social behavior of mice. It was used to evaluate the long-term impact of JH and PBA on social abilities, first in the presence of an unknown mouse (social approach phase), then in the presence of two mice, one familiar and one new unknown mouse (social memory phase). For this purpose, a rectangular device (90 × 43 cm) containing two multi-perforated cylinders (h = 13 cm, d = 8 cm) placed equidistantly from the edges of the device (22 cm from edges) was used. Only the social behavior of the tested mice was measured. At P30, the tested mouse was placed alone in the device with two empty cylinders for 5 min for free exploration. Then, the social approach phase began when a “Stranger 1” mouse was introduced into one cylinder for 5 min. Both times spent by the tested mouse in contact with “Stranger 1” and in contact with the empty cylinder were measured. Twenty-four hours after, the second phase of the test, social memory, began. The tested mouse was replaced in the device for 5 min in the presence of both “Stranger 1” in the same cylinder as the day before and “Stranger 2” in the other cylinder. “Stranger 1” in the social approach phase becomes the “familiar” mouse in this social memory phase of the test. Interactions durations with the “familiar” mouse and with “Stranger 2” were measured [41].

#### 4.7.2. Balance Beam Test (P32; Figure 11)

This test essentially examines the ability of the animal to remain upright and to walk on an elevated and relatively narrow beam without falling onto the cushioned pads below or slipping to one side of the beam. It assesses motor skills, in particular fine motor coordination and balance. Mice had to cross a fine wooden beam (66 cm long and 0.6 cm wide). For motivational purposes, the home cage was placed at the end of the beam. The crossing time and the number and type of imbalances (normal, jump, crab, hesitation, and imbalance) were assessed [42]. This test gives information regarding the integrated function of cortical arousal, cerebral locomotor control, and neuromuscular function.

#### 4.7.3. Foot-Fault Test (P33; Figure 12)

This test allows evaluation of skilled walking as the ability to adapt gait according to the environmental context. Gait is influenced by the temporal and spatial integration of the cognitive and neuromusculoskeletal neural systems [43]. It mainly mobilizes the extrapyramidal tracts. To perform the test, mice had to cross along a horizontal ladder with irregularly spaced metal rungs, adjusting their steps. For motivational purposes, the home cage was placed at the opposite end of the ladder. The mouse made an error when one of its paws fell or slid between two bars. The duration and number of errors for each of the two runs were analyzed, and then total duration and total number of errors were calculated.

#### 4.7.4. Novel Object Recognition Test (P34; Figure 14)

This test is used to investigate mice learning and memory abilities and is based on their natural propensity for novelty. For this purpose, the first day, the mouse explored for 10 min a cage without litter (43 × 27 × 20 cm) in which two unknown identical objects were glued (2 cones: 1 × 6 cm). The following day, one of the two cones was replaced by an unknown rectangular object (2 × 1 × 6 cm). During the 5 min test, the interaction duration with each of the two objects was measured. Then, the preference index was calculated as follows:

Preference index = ((time exploring the novel object−time exploring the familiar object)/total exploration time) × 100 [44].

**Figure 14:**
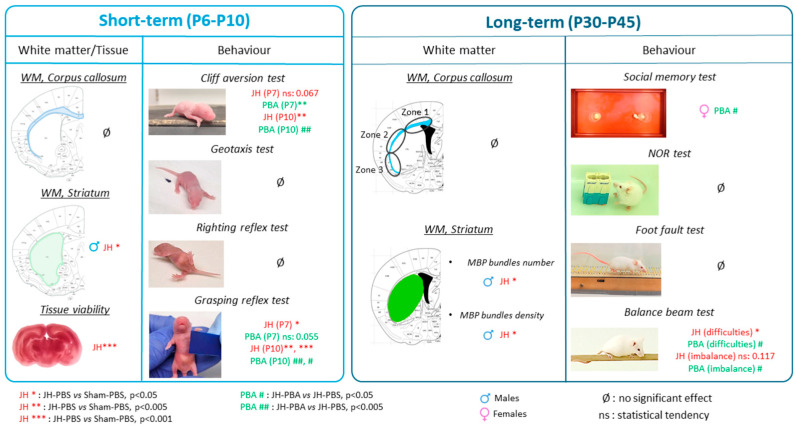
Summary of main results.

### 4.8. Statistical Analysis

Statistical analyses were performed in R (version 4.5.1) and graphs were produced with Graphpad Prism 9 software, San Diego, CA, USA. The rstatix library was used to conduct these analyses. The normality of the data and residuals was assessed using the shapiro_test() function. The homogeneity of variances was assessed using the levene_test() function. If the hypotheses were validated, a three-way ANOVA (Sex, Treatment, Surgery) was performed using the anova_test() function, followed by a Tukey post hoc test using the TukeyHSD() function. Otherwise, a Kruskal–Wallis test was performed using the krusksal_test() function, followed by a Dunn post hoc test using the dunn_test() function. Indeed, potential sexual differences were systematically investigated [5]. Results from males and females are presented in separated graphs when there was a significant effect of the Sex factor in the ANOVA or in the Kruskal–Wallis analysis. If not, sexes were pooled and a two-way ANOVA (Treatment, Surgery) was performed, followed by a Tukey post hoc test. Otherwise, in absence of normality, a Kruskal–Wallis test was performed, followed by a Dunn post hoc test. To analyze the distribution of MBP bundle area in the whole striatum, a Chi-square test was performed. No mouse was excluded, as there is a natural variability between individuals. The person manipulating the mice was aware of their group allocation in order to ensure that mice took the test sequentially. Video analysis of one test was performed by one person. Statistical analysis was conducted by another person. The significance level was set at 0.05. *: *p* < 0.05; **: *p* < 0.01; ***: *p* < 0.001 as compared to Sham-PBS mice. #: *p* < 0.05; ##: *p* < 0.01; ###: *p* < 0.001 as compared to JH-PBS mice.

## Data Availability

The original contributions presented in this study are included in the article/Supplementary Materials. Further inquiries can be directed to the corresponding author.

## Supplementary Materials

The following supporting information can be downloaded at: https://www.mdpi.com/article/10.3390/ijms262412099/s1.

## Abbreviations

The following abbreviations are used in this manuscript:

CC	Corpus callosum
JH model	Jugular Hypoxia model
MBP	Myelin basic protein
NA	Not applicable
NOR test	Novel object recognition test
OL	Oligodendrocyte
P5	Postnatal day 5
PA	Perinatal asphyxia
PBA	Sodium Phenylbutyrate
PDGFRa	Platelet-derived growth factor receptor alpha
WMI	White matter injury
