# Supplementary material for: Short- and Long-Term Effects of Sodium Phenylbutyrate on White Matter and Sensorimotor and Cognitive Behavior in a Mild Murine Model of Encephalopathy of Prematurity
Source: Int J Mol Sci. 2025 Dec 16;26(24):12099. doi: 10.3390/ijms262412099 (PMC12732855; doi:10.3390/ijms262412099)
Supplement: Supplementary file 1 [file ijms-26-12099-s001.zip › ijms-3991127-supplementary.pdf]

Supplementary data:

- Figure S1

-Table S1

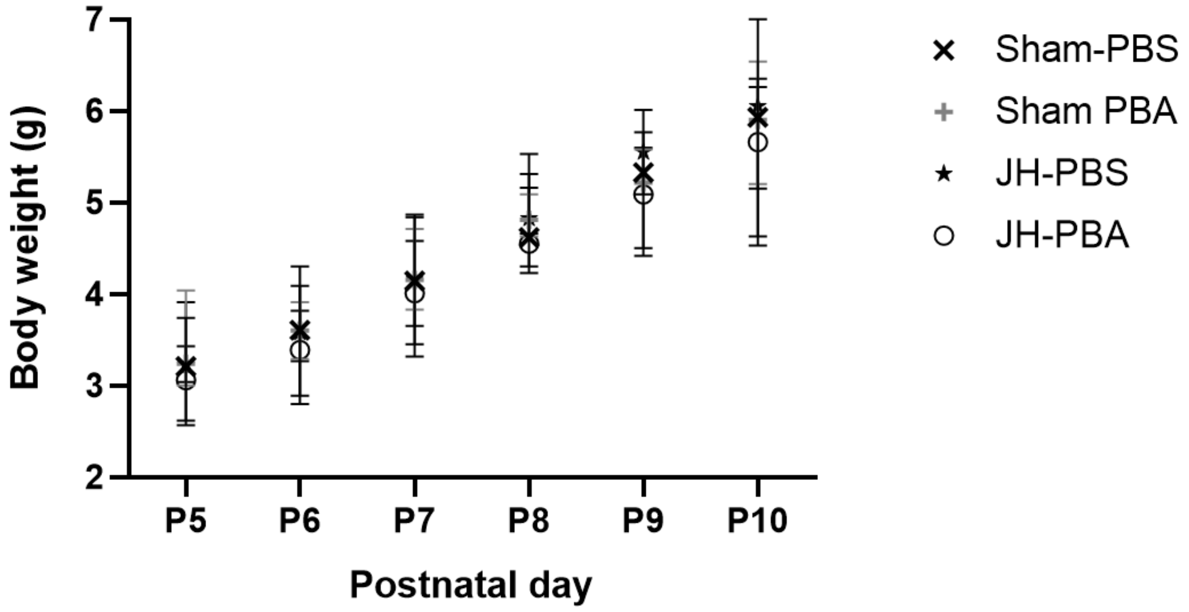

Figure S1: Effects of neonatal JH and/or PBA on pups' weight gain from P5 to P10.

The sexes are pooled. Data are expressed as median ± extreme values. *n*=6-19 mice/group. A three-way ANOVA test (Sex x Treatment x Surgery) was performed followed by a Tukey post-hoc test. Statistical details are provided in Table S1.

Table S1: Statistical details

Figure, Exp.	Details	Age	F(K-W test)	p	mice/group	compared groups	p
**Fig. 1**							
**PDGFRa density**	corpus callosum	P6	4.96	0.175	6-7		
	striatum, M		9.45	0.024 *		JH-PBS vs Sham-PBS	0.015 *
	striatum, F		5.41	0.015 *			ns
**Fig. 2**							
**TTC**	brain	P10	22.1	<0.001 ***	8-12	JH-PBS vs Sham-PBS	<0.001 *
**Fig. 3**							
**Grasping**	right rear	P6	6.46	0.091	11-19		
	left rear	P6	2.74	0.434			
	right rear	P7	12.9	0.005 **		JH-PBS vs Sham-PBS	0.029 *
						JH-PBA vs Sham-PBA	0.055 ns
	left rear	P7	11.8	0.008 **		JH-PBS vs Sham-PBS	0.008 **
	right front	P10	3.91	0.271			
	left front	P10	3.91	0.271			
	right rear	P10	22.4	<0.001 ***		JH-PBS vs Sham-PBS	<0.001 ***
						JH-PBA vs Sham-PBA	0.001 **
	left front	P10	15.9	0.001 **		JH-PBS vs Sham-PBS	0.003 **
						JH-PBA vs Sham-PBA	0.039 *
**Fig. 4**							
**Cliff**		P6	5.36	0.148	11-19	JH-PBS vs Sham-PBS	0.067 ns
		P7	16.3	<0.001 ***		JH-PBA vs Sham-PBA	0.001 **
						JH-PBS vs Sham-PBS	0.002 **
		P10	18	<0.001 ***		JH-PBA vs Sham-PBA	0.001 **
**Fig. 5**							
**Geotaxis**		P6	2.79	0.426	11-19		
		P7	0.585	0.9			
**Fig. 6**		P6	5.15	0.161	11-19		
**Righting**		P7	3.96	0.266			
**Fig. 7**							
**MBP Bundles density**	whole CC	P45	6.86	0.077 ns	6-8		
	zone 1 CC		4.85	0.183			
	zone 2 CC		2.68	0.444			
	zone 3 CC		0.514	0.916			

Figure, Exp.	Details	Age	F(K-W test)	p	mice/group	compared groups	p
**Fig. 8**					6-8		
**MBP Bundles density**	whole str, M	P45	9.03	0.029 *		JH-PBS vs Sham-PBS	0.034 *
	whole str, F		6.19	0.103			
	ventral str, M		8.91	0.031 *		JH-PBS vs Sham-PBS	0.023 *
	ventral str, F		4.33	0.228			
			**Chi-square:**				
	distribution		X^2= 8,653	0,47			
**Fig. 9A**							
**Social approach**	stranger 1	P30	8.26	0.041 *	6-9		n.s.
	cylinder		3.68	0.298			
**Fig. 9B**							
**Social memory**	familiar, M	P31	3.49	0.341	6-9		
	familiar, F		11.28	<0.001 ***		JH-PBA vs Sham-PBA	0.016 #
	stranger 2		2.99	0.393			
**Fig. 10**							
**Beam**	crossing (s)		4.162	0.244	10-16		
	difficulties, nb		11.13	0.010 *		JH-PBS vs Sham-PBS	0.038 *
						JH-PBA vs Sham-PBA	0.010 #
	imbalances, nb		9.21	0.027 *		JH-PBS vs Sham-PBS	0.117 ns
						JH-PBA vs Sham-PBA	0.024 #
**Fig. 11**							
**Foot fault**	crossing 1, nb	P33	3.68	0.298	8-16		
	crossing 2, nb		5.61	0.132			
	crossing 1+2, nb		6.19	0.103			
	crossing 1 (s)		2.32	0.508			
	crossing 2 (s)		4.05	0.256			
	crossing 1+2 (s)		2.42	0.489			
**Fig. 12**							
**NOR**	unknown (s)	P34	0.292	0.962	8-17		
	familiar (s)		1.42	0.7			
	preference		1.06	0.786			
**Suppl Fig. 1**			**F(ANOVA):**				
**Body weight**		P5	1.584	0.234	6-19		
		P6	0.702	0.42			
		P7	0.127	0.728			
		P8	0.813	0.387			
		P9	1.507	0.245			
		P10	1.249	0.288			
